# The complete chloroplast genome of *Stryphnodendron adstringens* (Leguminosae - Caesalpinioideae): comparative analysis with related Mimosoid species

**DOI:** 10.1038/s41598-019-50620-3

**Published:** 2019-10-02

**Authors:** Ueric José Borges de Souza, Rhewter Nunes, Cíntia Pelegrineti Targueta, José Alexandre Felizola Diniz-Filho, Mariana Pires de Campos Telles

**Affiliations:** 1 Laboratório de Genética & Biodiversidade, Departamento de Genética, Instituto de Ciências Biológicas - UFG, Goiânia, 74690-900 Brazil; 2 Laboratório de Ecologia Teórica e Síntese, Departamento de Ecologia, Instituto de Ciências Biológicas - UFG, Goiânia, 74690-900 Brazil; 3 Escola de Ciências Agrárias e Biológicas, PUC-Goiás, Goiânia, Brazil

**Keywords:** Comparative genomics, Plant genetics

## Abstract

*Stryphnodendron adstringens* is a medicinal plant belonging to the Leguminosae family, and it is commonly found in the southeastern savannas, endemic to the Cerrado biome. The goal of this study was to assemble and annotate the chloroplast genome of *S. adstringens* and to compare it with previously known genomes of the mimosoid clade within Leguminosae. The chloroplast genome was reconstructed using *de novo* and referenced-based assembly of paired-end reads generated by shotgun sequencing of total genomic DNA. The size of the *S. adstringens* chloroplast genome was 162,169 bp. This genome included a large single-copy (LSC) region of 91,045 bp, a small single-copy (SSC) region of 19,014 bp and a pair of inverted repeats (IRa and IRb) of 26,055 bp each. The *S. adstringens* chloroplast genome contains a total of 111 functional genes, including 77 protein-coding genes, 30 transfer RNA genes, and 4 ribosomal RNA genes. A total of 137 SSRs and 42 repeat structures were identified in *S. adstringens* chloroplast genome, with the highest proportion in the LSC region. A comparison of the *S. adstringens* chloroplast genome with those from other mimosoid species indicated that gene content and synteny are highly conserved in the clade. The phylogenetic reconstruction using 73 conserved coding-protein genes from 19 Leguminosae species was supported to be paraphyletic. Furthermore, the noncoding and coding regions with high nucleotide diversity may supply valuable markers for molecular evolutionary and phylogenetic studies at different taxonomic levels in this group.

## Introduction

The chloroplast, which is considered to have originated from free-living cyanobacteria through endosymbiosis, plays an essential role in photosynthesis and in many processes in plant cells^1–3^. In this evolutionary context, the chloroplast genome of angiosperms exhibit a highly conserved organization with a quadripartite structure, comprising two copies of inverted repeats (IRs), separated by large (LSC) and small (SSC) single-copy regions^4^.

The size of the circular chloroplast genome range between 120 and 160 kb in length^5^, but varies considerably both within and among plant families. For example, in the Geraniaceae, the size of the chloroplast genome ranges from 116,935 bp in *Erodium carvifolium*^6^ to 242,575 bp in *Pelargonium transvaalense* (Accession: NC_031206.1 unpublished). For Leguminosae, the size ranges from 120,289 bp in *Lathyrus odoratus* (Accession: NC_027150.1 unpublished) up to 178,887 in *Pithecellobium flexicaule*^7^. The variations in size can be attributed mostly to the expansion, contraction or loss of IRs, as well as variation in length of intergenic spacers^5,8^.

Most angiosperm chloroplast genome usually contain 100–130 distinct genes, comprising of 80–90 protein coding genes and approximately 30 transfer RNA (tRNA) genes and four ribosomal RNA (rRNA) genes^9,10^. The IR region comprises a duplicated set of tRNA and rRNA genes, whereas the single copy regions mostly consists of protein-coding genes involved in cell functions, which include components of the photosynthetic machinery (such as photosystem I (PSI), photosystem II (PSII), the cytochrome b6/f complex, and the ATP synthase), transcription, and translation. The two IRs are identical in their nucleotide sequence, so that every gene contained within them is present in two copies per genome which only differ in their relative orientation^9–11^.

The gene order and content of chloroplast genomes are generally highly conserved along plant evolution and the substitution rates are much lower than that of the nuclear genome^12^. This fact, coupled with the non-recombinant nature and maternal inheritance in most angiosperms, makes plant chloroplasts genomes valuable sources of genetic markers for analyzing evolutionary relationships at multiple scales, ranging from short-term phylogeographic patterns up to phylogenetic relationships among large clades^13,14^.

The first complete chloroplast genomes were determined over 30 years ago, for *Nicotiana tabacum*^15^ and *Marchantia polymorpha*^16^. However, because the time and cost associated with the conventional Sanger sequencing, the reconstruction of complete chloroplast genome was impractical for non-model species. More recently, with the advent of next-generation sequencing technology, whole genome sequencing has increased dramatically^17^. This offers an alternative way to obtain chloroplast genome based on downstream bioinformatics pipelines that allows distinguishing plastid reads from nuclear and mitochondrial reads^18^. Currently, approximately 1,654 eudicotyledons chloroplast genomes have been sequenced and deposited in the NCBI Organelle Genome, out of which 114 belong to the legume family (Leguminosae) and 19 to Caesalpinioideae subfamily.

The Leguminosae is the third-largest angiosperm family, with approximately 751 genera and ca. 19,500 species^19,20^. The Leguminosae was divided into three sub-families, the Caesalpinioideae, Mimosoid and Papilionoideae^20^. However, a new classification of the legumes has been proposed by The Legume Phylogeny Working Group^21^. They used a *mat*K gene-based phylogeny and a wide Leguminosae sample (~90% of genera) to propose a new family organization consisting in six sub-families: Caesalpinioideae, Cercidoideae, Detarioideae, Dialioideae, Duparquetioideae and Papilionoideae^21^. The traditional subfamily Mimosoid is now recognized as a distinct ‘mimosoid clade’ nested in the reassigned Caesalpinioideae^21^. Within the ‘mimosoid clade’, the genus *Stryphnodendron* Mart. includes approximately 21 species and two subspecies, mainly found in the South-American neotropical savannas^22^. Recently phylogenetic analysis demonstrated that the genus *Stryphnodendron* are not monophyletic^23^, clustering with the monospecific genus *Microlobius* inside the *Piptadenia* group^23^.

The *S. adstringens*, popularly known as “barbatimão”, is a common tree in the Brazilian Savanna. It’s a small, hermaphroditic, deciduous tree with a rough, light-colored, thick, tortuous trunk. It can reach 4–5 meters tall and the trunk can be 20–30 cm in diameter. The leaves alternate between composed and binary. Flowering occurs in September^24,25^ and the fruits are sessile, thick and fleshy, linear, oblong, light brown in color, 10 cm long, producing many brown seeds. The stem bark of this plant is used, popularly, in the treatment of several diseases because of it´s anti-inflammatory, antimicrobial and antiulcerogenic properties^26–29^. These effects are directly correlated to the presence of high concentrations of tannin into the barks^30^.

The goal of this study was to assemble the chloroplast genome of *S. adstringens* from whole genome sequence data, reporting the annotation and its structural characterization providing new genomic resources for this species. We also used a phylogenetic analysis to evaluate the sequence divergence in chloroplast regions of *S. adstringens* when compared with other known species of the mimosoid clade.

## Materials and Methods

### DNA extraction and chloroplast genome sequencing

Fresh young leaves of *S. adstringens* were collected in Niquelândia, Goiás, Brazil (*Sisgen Registration: A4EE2BE*). Total genomic DNA was extracted using a CTAB protocol^31^. DNA quality was evaluated using horizontal electrophoresis with 1% agarose gel. In addition, DNA was quantified through fluorometry using Qubit 2.0 (Life Technologies). Genomic library preparation was performed using a Nextera DNA Sample Preparation Kit (Illumina, San Diego, USA). The resulted library was sequenced using the HiSeq2500 platform and V4 SBS kit (Illumina) on a single lane in paired-end mode (2 × 100 bp) at the University of São Paulo (Escola Superior de Agricultura Luiz de Queiroz da Universidade de São Paulo) in Piracicaba, Brazil.

### Chloroplast genome assembly and annotation

Paired-end Illumina raw reads were filtered and trimmed using Trimmomatic V.0.36^32^ using the ILLUMINACLIP: NexteraPE-PE.fa:2:30:10 for adapter trimming, a sliding window of 10 base pairs with a minimum average quality score of 20 (SLIDINGWINDOW:10:20), and a minimum length of 40 bp (MINLEN:40).

The chloroplast genome of *S. adstringens* was reconstructed using a combination of *de novo* and reference-guided assemblies. To obtain the *de novo* chloroplast genome assembly, the paired-end sequence reads were mapped to five Mimosoid plastomes using Bowtie2 v.2.3.4.1^33^ to exclude reads of nuclear and mitochondrial origins (*Adenanthera microsperma* Teijsm. & Binn. [accession no. NC_034986], *Dichrostachys cinerea* (L.) Wight & Arn. [accession no. NC_035346], *Leucaena trichandra* (Zucc.) Urb. [accession no. NC_028733], *Parkia javanica* (Lam.) Merr. [accession no. NC_034989], *Piptadenia communis* Benth. [accession no. NC_034990]). The obtained putative chloroplast reads were then used for *de novo* assembly using SPAdes 3.6.1 with iterative K-mer sizes of 55, 69 and 87^34^. Reference guided assembly was performed with YASRA 2.32^35^ using *Piptadenia communis* Benth. as reference chloroplast genome. Contigs with coverage below than 10x were eliminated. The remaining *de novo* contigs were merged with reference-guided contigs in Sequencher 5.4.6 (Genecodes, Ann Arbor, Michigan, USA) based on at least 20 bps overlap and 98% similarity. Any discrepancies between *de novo* and reference-guided contigs were corrected by searching the high quality read pool using the UNIX ‘grep’ function. A “genome walking” technique, using the Unix “grep” function, was used to find reads that could fill any gaps between contigs that did not assemble in the initial set of analyses. Assembly curation was performed by aligning sequencing reads on the chloroplast using the Bowtie2 program. Sequencing depth was measured using the samtools platform (samtools.sourceforge.net/). Additionally, we also compared the position of the chloroplast genome regions of *S. adstringens* related species in circle alignment graphs made with the Circus program (http://circos.ca/).

Annotation of the chloroplast genome was performed using Verdant^36^ and Dual Organellar Genome Annotator-DOGMA^37^, coupled with manual correction of start and stop codons and intron/exon boundaries. Transfer RNA (tRNA) genes were identified with DOGMA and the tRNAscan-SE program ver. 2.0^38^ in organellar search mode with default parameters. The circular chloroplast genome map was drawn using OrganellarGenomeDRAW (OGDRAW)^39^. The codon usage analysis was performed in the web server Bioinformatics (https://www.bioinformatics.org/sms2/codon_usage.html).

### Characterization of repeat sequences

The sizes and locations of forward, reverse, palindromic and complementary repeats in the *S. adstringens* chloroplast genome were determined by REPuter^40^ with a minimal size of 30 bp, hamming distance of 3 and over 90% identity. Simple sequence repeats (SSRs) were detected using the microsatellite identification tool MISA (available online: http://pgrc.ipk-gatersleben.de/misa/misa.html). The minimum number of SSRs was set to ten repeat units for mononucleotide, five repeat units for dinucleotide, four repeat units for trinucleotide and three repeat units for tetra-, penta- and hexanucleotide.

### Nucleotide Diversity and Synonymous (Ks) and non-synonymous (Ka) substitution rate analysis

The complete chloroplast genome sequence of *S. adstringens* was compared with the chloroplast genome sequences of five Mimosoid chloroplast genomes used for assembling. To assess the complete nucleotide diversity (*Pi*) among the complete chloroplast genome of the six species, the complete chloroplast genome sequences were aligned using MAFFT aligner tool^41^, and manually adjusted with Bioedit^42^. We then performed a sliding window analysis to calculate the nucleotide variability (*Pi*) values using DnaSP 6^43^ with window lenght of 600 bp and step size of 200 bp. The 77 protein-coding genes were extracted and aligned separately using MAFFT to estimate the synonymous (Ks) and non-synonymous (Ka) substitution rates. The Ka/Ks for each gene were estimated in DnaSP 6.

### Comparative analysis of genome structure

The mVISTA program was applied to compare the complete chloroplast genome of *S. adstringens* against the whole chloroplast genome of the five mimosoid species using the shuffle-LAGAN mode^44^. The annotated *S. adstringens* chloroplast genome was used as reference. The expansion and contraction of the IR regions at junction sites between the six mimosoid species were verified and plotted using IRscope^45^.

### Phylogenetic analyses

Seventy-three protein-coding genes were recorded from 19 species within the Leguminoseae – Caesalpinioideae, as well as from two outgroups (*Cucumis sativus* L. and *Fragaria vesca* L.). All genes sequences were obtained from GenBank (see Supplementary Table S6 for accession numbers). The *accD*, *ycf*1, *rps16*, *rps12* genes were not considered for phylogenetic analysis as they were not present in all chloroplast genomes among the species under analysis.

The nucleotide sequences were aligned using MAFFT^41^ with default parameters. The Akaike Information Criterion (AIC) in JModelTest v2.1.10 was used to determine the best-fitting model of molecular evolution for each gene^46^ (models selected can be seen in Supplementary Table S7). The alignments from the 73 protein-coding genes were concatenated and a Bayesian inference was performed using BEAST v1.10.1^47^ at the XSEDE Teragrid of the CIPRES science gateway^48^ (available online: www.phylo.org). The Markov chain Monte Carlo (MCMC) was set to run 50.000.000 generations and sampled every 1.000 generations, under a strict clock approach using the Yule speciation tree prior with the evolutionary models. The Convergence of parameters during MCMC runs were assessed by their Effective Sample Size (ESS) > 200 using TRACER v1.7.1^49^. The phylogenetic tree was annotated as a maximum clade credibility tree using TREEANNOTATOR v.2.5.0 (part of the BEAST package), with burn in of 20%. The final tree was produced using FigTree v.1.4.3 (http://tree.bio.ed.ac.uk/software/figtree/).

## Results and Discussion

### Genome assembly and annotation

A total of 563,117,260 raw Illumina paired-end reads from the *S. adstringens* genome were generated and filtered against Mimosoid chloroplast genomes. After trimming adapters, low quality bases, and mapping the reads to the reference set, a total of 10,549,708 reads were used to assemble the chloroplast genome. The filtered reads were assembled into 57 contigs with at least 10x of coverage with a total length of 271,468 bp and an N50 of 10,881. The reference guided assembly produced 64 contigs with an N50 of 3,938 (half of the assembly contained at least 3,938 bp). The final assembly from both approaches resulted in a single chromosome and was benchmarked based on the distribution of sequencing coverage by base. After that, for validation, a set of 18,937,635 raw paired-end Illumina reads were well aligned in the chloroplast genome. The average sequencing depth was 11,470X , with a standard deviation of 3,720 (median: 12,009; mode: 12,448; minimum: 29 and maximum: 72,245) (Supplementary Fig. 1A,B). The pairwise alignments with species close related to *S. adstringens* showed a high conservation of the general structure of the chromosome and correct arrangement of the chloroplast regions validating the proposed genome (Supplementary Fig. 2A–D). The sequence of the chloroplast genome was deposited in GenBank (accession number: MN196294).

**Figure 1:**
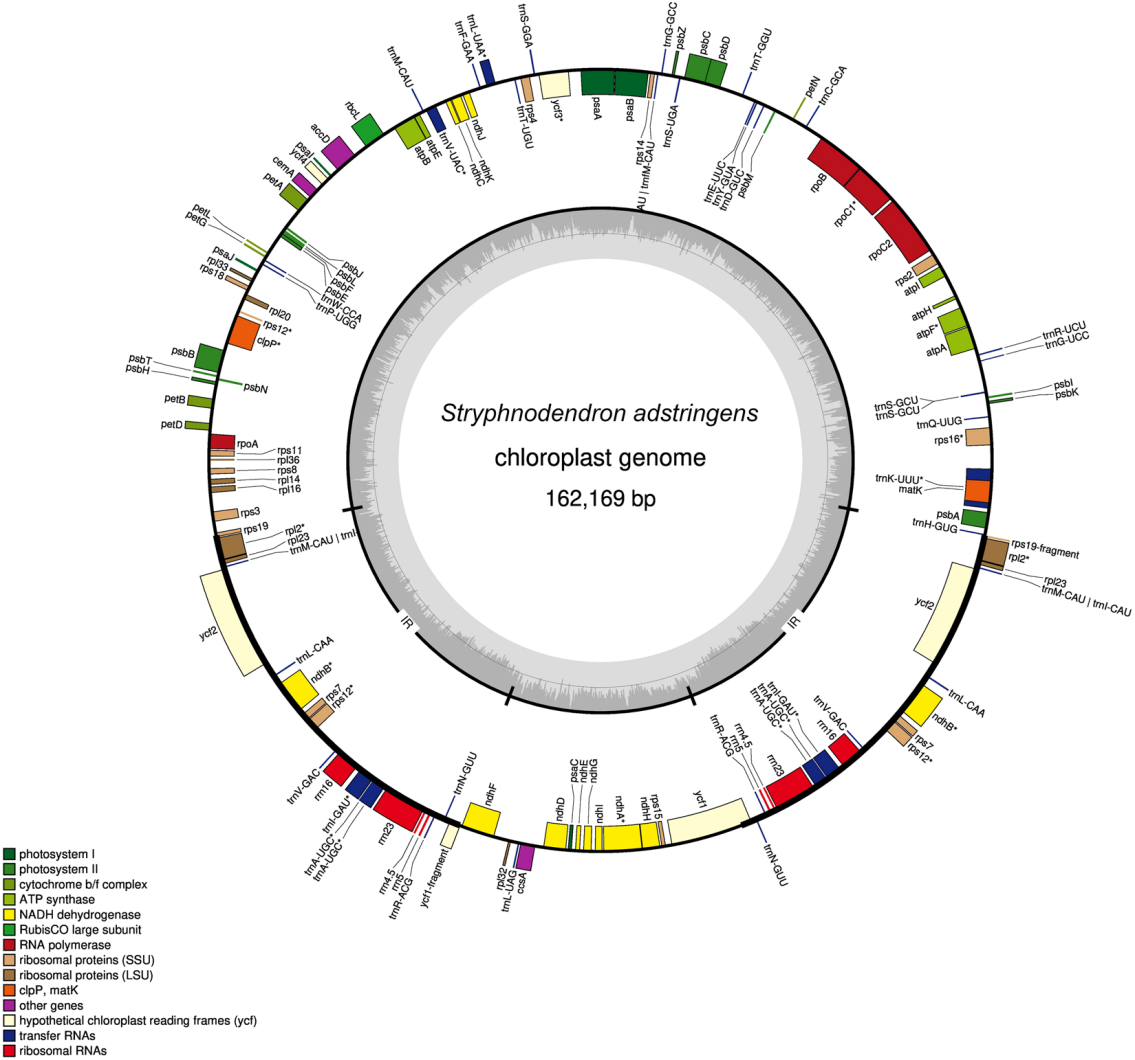
Gene map of the *S. adstringens* chloroplast genome. The genes drawn outside and inside of the circle are transcribed in clockwise and counterclockwise directions, respectively. Genes were colored based on their functional groups. The inner circle shows the quadripartite structure of the chloroplast: small single copy (SSC), large single copy (LSC) and a pair of inverted repeats (IRa and IRb). The gray ring marks the GC content with the inner circle marking a 50% threshold. Asterisks mark genes that have introns.

The complete chloroplast genome of *S. adstringens* was assembled with a total of 162,169 bp in size, divided in four regions, which included a large single-copy (LSC) region of 91,045 bp, a small single-copy (SSC) region of 19,014 bp, separated by two inverted repeat (IR) regions of 26,055 bp each (Fig. 1; Table 1). Analogous to most angiosperms, the *S. adstringens* chloroplast genome comprises a single circular molecule with a quadripartite structure^4^ and it is similar in size from other species from the Mimosoid clade (Leguminosae; Table 1; see also^7^).

**Table 1:** Chloroplast genome information from sampled Mimosoid species and the newly assembled *S. adstringens*.

Species	Size (bp)	LSC (bp)	SSC (bp)	IR (bp)	GC (%)	Protein	RNA
*Adenanthera microsperma*	159,389	88,577	18,756	26,028	36.5%	77	34
*Dichrostachys cinerea*	161,240	90,430	18,526	26,142	35.9%	77	34
*Leucaena trichandra*	164,692	93,690	18,890	26,056	35.6%	77	34
*Parkia javanica*	161,681	91,093	18,574	26,007	35.9%	77	34
*Piptadenia communis*	162,552	91,517	18,941	26,047	35.9%	77	34
*Stryphnodendron adstringens*	162,169	91,045	19,014	26,055	35,9%	77	34

LSC Large Single Copy, SSC Small Single Copy, IR Inverted Repeat.

The GC content of the *S. adstringens* chloroplast genome was 35.9%, which is also consistent with other Mimosoid, whose plastomes ranged from 35.6 to 36.5% overall GC content (Table 1; see also^7^). Among the LSC, SSC and IR regions, the highest GC content was found in the IR regions (42.7%), while the GC content of LSC and SSC was 33.3% and 30.0%, respectively (Supplementary Table S1). The high GC content in the IR regions are mainly due to high GC contents of the four ribosomal RNA (rRNA) genes, *rrn23*, *rrn16*, *rrn5*, *rrn4.5*, with 55.3%, 56.4%, 52.9% and 50%, respectively, that are located in this region.

The assembled chloroplast genome contained 111 different genes, with 77 protein-coding genes, 30 transfer RNA (tRNA) and 4 ribosomal RNA genes (rRNA) (Fig. 1; Table 2). A total of nine protein-coding genes and 6 tRNAs genes contained a single intron, whereas three genes (*rps12*, *clpP* and *ycf3*) exhibit two introns each (Supplementary Table S2). The *rps12* gene was predicted to be trans-spliced, with the 5′ end located in the LSC region and the duplicated 3′ end in the IR region. The *trnK-UUU* has the largest intron encompassing the *matK* gene, with 2,558 bp, whereas the intron of *trnL-UAA* is the smallest (513 bp).

**Table 2:** List of genes in the chloroplast genome of *S. adstringens*.

Category	Gene groups	Name of genes
Self-replication	Large subunit of ribosomal proteins	*rpl2*^1,2^, *rpl14*, *rpl16*^1^, *rpl20*, *rpl23*^2^, *rpl32*, *rpl33*, *rpl36*
	Small subunit of ribosomal proteins	*rps2*, *rps3*, *rps4*, *rps7*^2^, *rps8*, *rps11*, *rps12*^*1*,2^, *rps14*, *rps15*, *rps16*^1^, *rps18*, *rps19*
	DNA-dependent RNA polymerase	*rpoA*, *rpoB*, *rpoC1*^1^, *rpoC2*
	Ribosomal RNA genes	*rrn4.5*^2^, *rrn5*^2^, *rrn16*^2^, *rrn23*^2^
	Transfer RNA genes	*trnA-UGC*^1,2^, *trnC*-*GCA*, *trnD*-*GUC*, *trnE-UUC*, *trnF*-*GAA*, *trnfM*-*CAU*, *trnG*-*UCC*^1^, *trnG-UCC, trnH*-*GUG*, *trnI*-*CAU*^2^, *trnI*-*GAU*^1,2^, *trnK*-*UUU*^1^, *trnL*-*CAA*^2^, *trnL*-*UAA*^1^, *trnL*-*UAG*, *trnM*-*CAU*, *trnN*-*GUU*^2^, *trnP*-*UGG*, *trnQ*-*UUG*, *trnR*-*ACG*^2^, *trnR*-*UCU*, *trnS*-*GCU*, *trnS*-*UGA*, *trnS*-*GGA*, *trnT*-*UGU*, *trnT*-*GGU*, *trnV*-*UAC*^1^, *trnV*-*GAC*^2^, *trnW*-*CCA*, *trnY*-*GUA*
Photosynthesis	Photosystem I	*psaA*, *psaB*, *psaC*, *psaI*, *psaJ*
	Photosystem II	*psbA*, *psbB*, *psbC*, *psbD*, *psbE*, *psbF*, *psbH*, *psbI*, *psbJ*, *psbK*, *psbL*, *psbM*, *psbN*, *psbT*, *psbZ*
NADH dehydrogenase	NADH dehydrogenase	*ndhA*^1^, *ndhB*^1,2^, *ndhC*, *ndhD*, *ndhE*, *ndhF*, *ndhG*, *ndhH*, *ndhI*, *ndhJ*, *ndhK*
	Cytochrome b/f complex	*petA*, *petB*^1^, *petD*^1^, *petG*, *petL*, *petN*
	ATP synthase	*atpA*, *atpB*, *atpE*, *atpF*^1^, *atpH*, *atpI*
	RubisCo large subunit	*rbcL*
Other genes	Maturase K	*matK*
	Envelope membrane protein	*cemA*
	Subunit of acetyl-CoAcarboxylase	*accD*
	C-type cytochrome synthesis gene	*ccsA*
	Protease	*clpP* ^1^
	Conserved hypothetical chloroplast open reading frames	*ycf1*, *ycf2*^2^, *ycf3*^1^, *ycf4*

1 – Gene with introns.

2 – Gene completely duplicated in the inverted repeat.

Codon usage analysis performed using 77 protein coding gene sequences identified a total of 20,986 codons in the *S. adstringens* chloroplast genome (Table 3). Most identified codons are coders for amino acid leucine (2,227 codons, ~ 10.6% of the total number of codons) and the most abundant codon was TTA (33% of codons encoding leucine). The codons encoding the amino acid cysteine were identified as the least abundant in the *S. adstringens* chloroplast genome (256 codons, ~1.2%). Moreover, only one codon was identified for the coding of methionine (ATG) and tryptophan (TGG) amino acids.

**Table 3:** Codon usage for *S. adstringens* chloroplast genome.

Amino Acid	Codon	Number	Fraction	Amino Acid	Codon	Number	Fraction
Ala	GCG	138	0.12	Leu	CTA	302	0.14
	GCA	321	0.27		CTT	462	0.21
	GCT	571	0.48		CTC	135	0.06
	GCC	168	0.14	Lys	AAG	245	0.24
Arg	AGG	134	0.11		AAA	759	0.76
	AGA	365	0.30	Met	ATG	504	1
	CGG	92	0.07	Phe	TTT	803	0.67
	CGA	282	0.23		TTC	397	0.33
	CGT	275	0.22	Pro	CCG	112	0.13
	CGC	87	0.07		CCA	249	0.29
Asn	AAT	737	0.78		CCT	341	0.39
	AAC	208	0.22		CCC	170	0.19
Asp	GAT	655	0.81	Ser	AGT	324	0.20
	GAC	149	0.19		AGC	97	0.06
Cys	TGT	186	0.74		TCG	145	0.09
	TGC	67	0.26		TCA	312	0.19
Gln	CAG	164	0.23		TCT	458	0.29
	CAA	559	0.77		TCC	267	0.17
Glu	GAG	264	0.25	Thr	ACG	113	0.11
	GAA	784	0.75		ACA	322	0.30
Gly	GGG	238	0.16		ACT	442	0.42
	GGA	593	0.39		ACC	188	0.18
	GGT	531	0.35	Trp	TGG	370	1
	GGC	152	0.10	Tyr	TAT	622	0.80
His	CAT	395	0.78		TAC	153	0.20
	CAC	112	0.22	Val	GTG	168	0.14
Ile	ATA	564	0.31		GTA	460	0.38
	ATT	933	0.51		GTT	434	0.36
	ATC	341	0.19		GTC	134	0.11
Leu	TTG	466	0.21	End	TGA	29	0.28
	TTA	727	0.33		TAG	21	0.21
	CTG	138	0.06		TAA	52	0.51

The duplicated IR of the *S. adstringens* chloroplast genome resulted in complete duplication of sixteen genes (including five protein-coding genes [*rpl2*, *rpl23*, *rps7*, *rps12* and *ndhB*], seven tRNAs [*trnA-UGC*, *trnI-CAU*, *trnI-GAU*, *trnL-CAA*, *trnN-GUU*, *trnR-ACG*, *trnV-GAC*], and all four rRNAs [*rrn23*, *rrn16*, *rrn5*, *rrn4.5*], see Table 2; Fig. 1) and parts of the 5′ end of *ycf1* and *rps19*. Of the remaining genes, the LSC region contained 59 protein-coding and 22 tRNA genes, while the SSC region contained 11 protein-coding and one tRNA gene. These results corroborate the findings of Wang *et al*. (2017), which all species belonging to tribe Mimoseae had canonical IRs, with a typical gene content and general organization^7^.

It is important to point out that the structure of the chloroplast genome in species from Leguminosae family are highly variable because of either expansion or contraction of the IR^7,50,51^. This is mainly caused by the gene transfer from single copy regions to inverted repeat and vice versa in the boundaries of IRs, during evolution^51^. With the exception of *Acacia ligulata*^52^, the legume plastomes of all species documented to date within the clade formed by *Ingeae* and *Acacia* species, have IRs ca. 13 kb larger, and a SSC correspondingly smaller, than other legumes^7,50^. In addition, the size variation of the plastid genome has been explained, at least in part, by the loss of one IR. However, the mechanisms that led to IR loss are still unknown. Examples of variation within the Leguminosae include the loss of the IR in the monophyletic group within the subfamily Papilionoideae (including *Trifolium*, *Pisum*, *Cicer*, *Medicago*, *Glycyrrhiza* and *Vicia*), known as the “inverted repeat lacking clade”^53,54^.

### Repeat sequences analysis

A total of 42 repeat structures, with lengths ranging from 30 bp to 128 bp, were detected in the *S. adstringens* chloroplast genome. They included 22 (52.38%) forward repeats, 18 (42.86%) palindromic repeats, and two (4.76%) reverse repeats (Supplementary Table S3), whereas none complementary structure was identified. The forward repeats ranged from 30 bp to 128 bp. The palindromic repeats were 30 bp to 60 bp, whereas the two reverse repeats were 30 bp and 38 bp (Supplementary Table S3). In the majority of Mimosoid species, the most abundant dispersed repeat identified were forward, then palindromic and the least was reverse^7^.

Among the 42 repeats, 64.29% are located in the LSC region, 14.29% in the SSC region and 16.67% in the IR region. Two repeats, which were 39 bp and 52 bp, located in the intron region of *ycf3* and *rpl16*, was found to repeat thrice (LSC/SSC/IR) and twice (LSC/SSC), respectively, as forward repeat. Most of the repeats (80.95%) were found in the intergenic spacer regions (IGS), whereas 19.05% were located in the introns (*ndhA*, *ycf3* and *rpl16*) and only two were located in coding region (*psaB* and *trnS-GCU*) (Supplementary Table S3).

**Figure 2:**
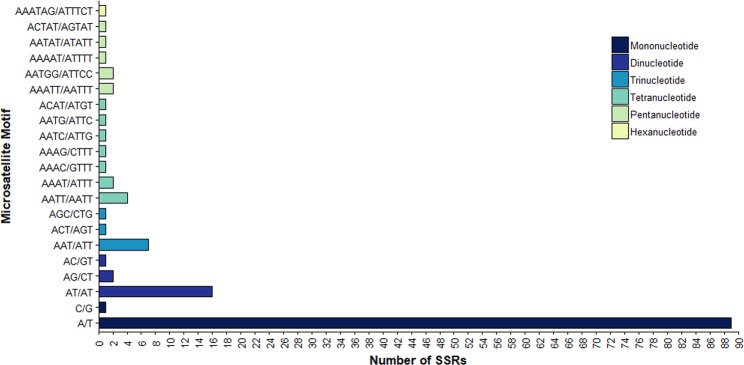
Number and type of simple sequence repeats in *S. adstringens* chloroplast genome.

A total of 137 SSRs were detected in the *S. adstringens* chloroplast genome, which were composed by a length of at least 10 bp and repeated 3 to 21 times. Among them, 90 (65.69%) were mono-repeats, 19 (13.87%) were di-repeats, nine (6.57%) were tri-repeats, 11 (8.03%) were tetra-repeats, seven (5.11%) were penta-repeats and one (0.73%) was hexa-repeat. The majority (89 or 98.89%) of the mononucleotide repeats consisted of A/T motifs, while only one was composed of a G/C motif. Likewise, most of the dinucleotides and trinucleotides were AT-rich, being composed of AT/TA (84.21%) and AAT/TTA (77.78%) repeats, respectively (Fig. 2). These results showed that the SSRs exhibit a strong AT bias, which is consistent with the observed in other Leguminosae species, such as *Vigna radiata*^55^, *Cajanus cajan*^10^, *Cajanus scarabaeoides*^10^ and *Arachis hypogaea*^56^.

Concerning genomic localization, among the 137 SSRs, 114 (83.21%) were found in the LSC region, 17 (12.41%) in the SSC region and six (4.38%) in the IR region (Supplementary Table S4). Most of these SSRs were located in intergenic regions (102 or 74.45%), while 17 (12.41%) were in the introns and 18 (13.14%) were in the protein-coding genes (Supplementary Table S4). The *ycf1* gene contained more SSRs than the other genes (Supplementary Table S4). In addition, the results found herein are in agreement with those from *Cajanus cajan*^10^, *Cajanus scarabaeoides*^10^, *Vigna radiata*^55^ and *Glycine* species^57^. The *ycf1* encodes a protein of approximately 1,800 amino acids. The *ycf1* located in the IR region is short and conserved, while the located in SSC region are extremely variable in seed plants^58,59^. Some studies reported that this region is the most variable locus for the design of primers, as well, as more variable than *matK* in many taxa, and thus suitable for molecular systematics at low taxonomic levels^58,60^.

### Nucleotide diversity and Ka/Ks ratio

The average nucleotide variability (*Pi*) among the five chloroplast genomes of Mimosoid species was estimated to be 0.01771, ranging from 0 to 0.172. The nucleotide variability was higher in the SSC (*Pi* = 0.02712) and LSC (*Pi = *0.02488), when compared to IR regions, which had a much lower nucleotide diversity (*Pi* = 0.00339). Similar results were obtained in the comparison of chloroplast genome sequences among *Aconitum* L. species, in which the average *Pi* in the IR region was 0.00146, whereas in the LSC and SSC regions the diversity estimates were 0.007140 and 0.008368, respectively^61^. Park *et al*. (2018) analyzed the nucleotide diversity in six *Ipomoea* L. chloroplast genome and also found that the IR regions were more conserved than the LSC and SSC regions, with average *Pi* values of 0.003 for IR and more than 0.006 for SSC and LSC regions^62^.

**Figure 3:**
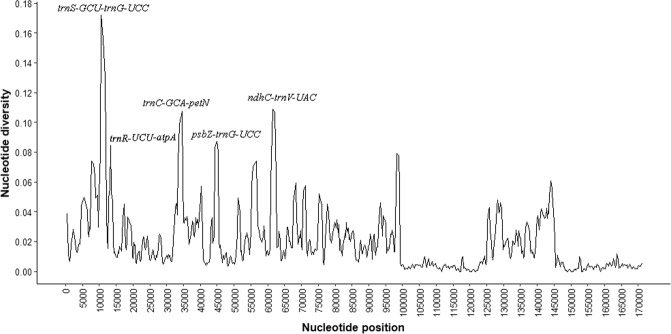
Sliding window analysis of Mimosoid chloroplast genomes.

Five regions (*trnS-GCU-trnG-UCC*, *trnR-UCU-atpA*, *trnC-GCA-petN*, *psbZ-trnG-UCC*, *ndhC-trnV-UAC*) showed high levels of nucleotide diversity, with *Pi* values > 0.8 (Fig. 3). All of these highly variable regions are found in intergenic spacer from LSC region. Liu *et al*. (2018) analyzed the nucleotide diversity among seven species from caesalpinioid legumes and reported five regions including *psbZ-trnG* (*trnT-trnL, rps3-rps19, rpl32* and *ycf1*) with higher *Pi* values, all with *Pi* > 0.12^63^. Highly variable regions among chloroplast genomes can be useful for phylogenetic reconstruction and may be used for further phylogenetic study of the genus *Stryphnodendron*.

**Figure 4:**
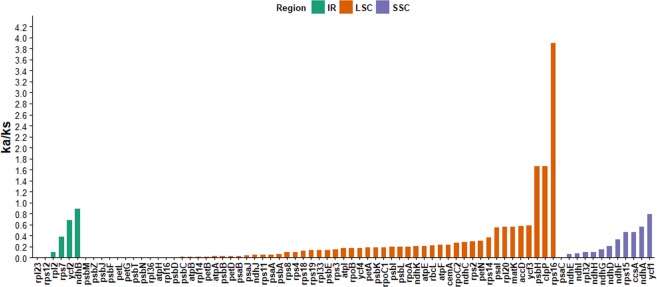
The Ka/Ks ratio of Mimosoid chloroplast genomes for individual genes.

The non-synonymous (Ka) to synonymous (Ks) rate ratio (Ka/Ks) was calculated for the 77 protein-coding genes in common across all six chloroplast genomes (Fig. 4 and Supplementary Table S5). The synonymous substitution does not change the amino acid within a peptide chain, whereas nonsynonymous substitution does. The *rpl32* gene associated with large subunit of ribosomal proteins had the highest synonymous rate, 0.09263, while the *ycf*1 gene with unknown functions, had the highest nonsynonymous rate, 0.03534.

The Ka/Ks ratio may indicate whether selective pressure is acting on a particular protein-coding gene. A Ka/Ks > 1 indicates that the gene is affected by positive selection, whereas Ka/Ks < 1 indicates that the gene is affected by negative selection or purifying selection. A value of 0, indicates the presence of neutral selection^64^. Concerning the different regions of chloroplast genomes, the Ka/Ks ratio were highest on average in the SSC region (0.2991) and lowest in the IR region (0.2856) and LSC region (0.2708). The lowest Ka/Ks ratio was observed for genes encoding subunits of ATP synthase, subunits of the cytochrome b/f complex, subunits of the large subunit of ribosomal proteins and subunits of photosystem II (Fig. 4; Supplementary Table S5).

Herein, the Ka/Ks ratio was calculated to be 0 for 13 genes, two inside the IR region (*rpl23*, *rps12*), ten in the LSC region (*rpl36*, *psbM, psbZ*, *psbJ*, *psbF*, *psbT*, *psbN*, *petL*, *petG*, *atpH*) and one in the SSC region (*psaC*) (Fig. 4). This occurred because the Ka or Ks is 0 or extremely low, thus Ka/Ks ratio could not be calculated^65,66^. Among the 77 protein-coding genes, Ka/Ks indicates purifying selection in 62 of them (Fig. 4; Supplementary Table S5). The Ka/Ks ration indicates positive selection for three genes analyzed, one of it is associated with small subunit of ribosomal proteins (*rps16*), the other is associated with Photosystem II (*psbH*) and the third with the *clpP* proteases (Fig. 4; Supplementary Table S5).

Liu *et al*. (2018) reported four genes with Ka/Ks ratio more than 1, indicating positive selection, *ndhD*, *ycf1*, *infA* and *rpl23* in caesalpinioid legumes^63^, whereas, Park *et al*. (2018) observed positive selection in three genes, *accD*, *cemA*, and *ycf2*, among six *Ipomoea* species^62^. In addition, Tian *et al*. (2018) reported only one gene (*rps12*) with positive selection among nine *Araceae* species^67^.

In addition, it was demonstrated that Leguminosae chloroplast have regions with accelerated mutation rates, including genic regions such as the *clpP* in Mimosoids and *rps16* in the IRLC clade^50,52,68^. The *rps16* gene encodes the ribosomal protein S16 and it is present in the chloroplast genome of the majority of higher plants. Moreover, a multiple gene-loss event of *rps16* was reported for various legumes lineages^69,70^. For instance, in the Leguminosae family, *Cicer arietinum*^71^, *Caragana rosea*^72^, *Phaseolus vulgaris*^73,74^, *Lupinus* species^70^ and *Mucuna macrocarpa*^75^ have lost this gene. As revealed in other studies, the multiple loss of the *rps16* was assumed to be a consequence of the dual targeting of the nuclear *rps16* copy to the plastid as well as the mitochondria, suggesting that the chloroplast-encoded *rps16* has already been silenced and has become a pseudogene by the nuclear-encoded *rps16*^69,70,76^.

### Comparative analysis of genome structure

**Figure 5:**
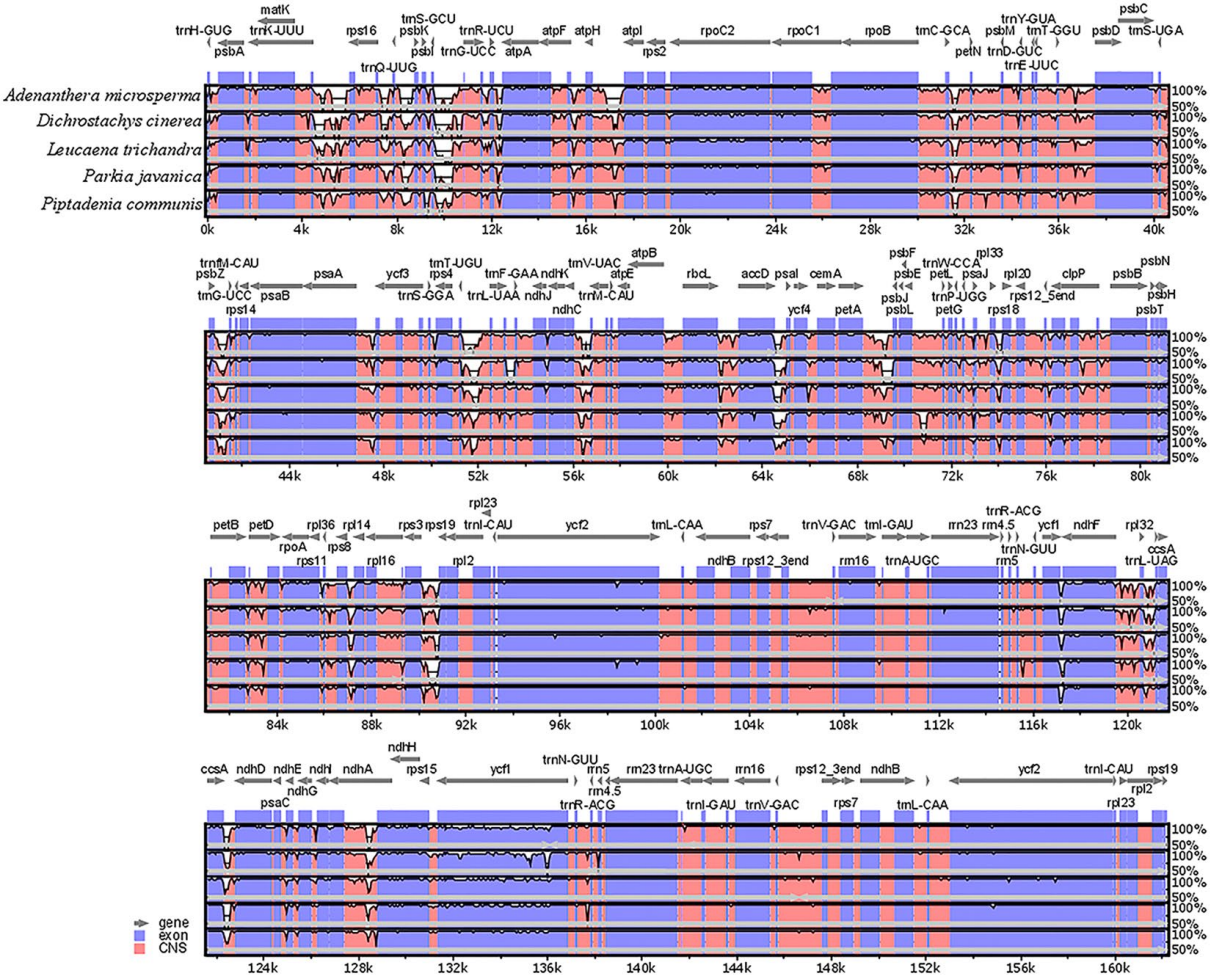
Visualization of genome alignment of six Mimosoid chloroplast genomes using *S. adstringens* as reference. The vertical scale indicates the percent of identity, ranging from 50% to 100%. Coding regions are marked in purple, and non-coding as red. The horizontal axis indicates the coordinates within the chloroplast genome.

The structural characteristics in chloroplast genome among Mimosoid species revealed that gene coding regions were more conserved than the noncoding regions, and IRs were more conserved than LSC and SSC regions (Fig. 5). This result is consistent with the pattern revealed in other Leguminosae species, such as *Glycine* species^77^ and species from Caesalpinioideae subfamily^7^. Additionality, it was also observed that the intergenic spacers regions between several pairs of genes varied greatly, for example, between *matK*-*rps16*, *rps16*-*psbK*, *trnS-GCU*-*trnG-UCC*, *atpH-atpI*, *trnC-GCA*-*petN*, *psbZ-trnG*, *trnT-UGU*-*trnL-UAA*, *ndhC*-*trnV-UAC* and *rps3*-*rps19*. Some of these intergenic spacer regions had also the highest level of nucleotide diversity.

**Figure 6:**
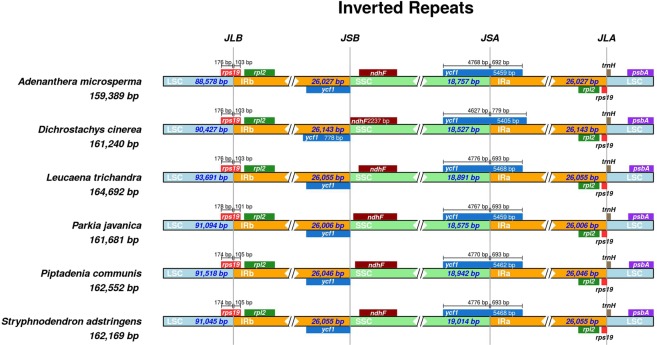
Comparison of the junction sites between the Long Single Copy (LSC, light blue), Short Single Copy (SSC, light green) and Inverted Repeat (IRa and IRb, orange) regions among the six Mimosoid chloroplast genomes. JLB (IRb/LSC), JSB (IRb/SSC) JSA (SSC/IRa) and JLA (IRa/LSC) denote the junction sites between each corresponding regions on the genome.

The expansion and contraction of the IR region and the single-copy boundary regions result in change in the position of the junction sites, which is considered as a primarily mechanism causing length variation of chloroplast genomes in higher plants^78^. The length of the IR regions was similar, ranging from 26,006 bp in *Parkia javanica* to 26,143 bp in *Dichrostachys cinerea* (Fig. 6). Wang *et al*. (2017) observed that species belonging to tribe Mimoseae had canonical IRs, whereas species belonging to tribes Ingae and Acacieae had much long IRs, as all of them experienced ca. 13 kb IR expansion into SSC previously reported by Dugas *et al*. (2015).

The endpoint of the Mimoseae JLA (Junction between IRa and LSC) was located upstream of the *rps19* and downstream of the *trnH-GUG*. Similar patterns were observed by Amiryousefi *et al*. (2018b) in Solanaceae chloroplast genomes^79^. The junction between IRb and SSC region (JSB) was located in the intergenic *ycf1*/*ndhF*, and the distance between the *ndhF* end to the junction of the IRb/SSC differs by 11 bp in *Dichrostachys cinerea* to 150 bp in *Adenanthera microsperma*. The junction between IRa and SSC (JSA) was located within the *ycf1* gene, and the fragment located at the IRa region ranged from 692 bp to 779 bp (Fig. 6). The gene *rps19* crossed the LSC/IRb region and the extent of the IR expansion to *rps19* slightly varies among the Mimoseae species ranging from 101 bp to 105 bp.

### Phylogenetic relationships

**Figure 7:**
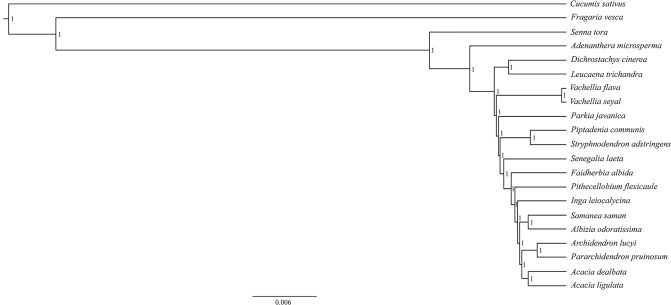
Maximum credibility tree reconstructed based on 73 conserved coding-protein genes from twenty-one species. All nodes of the phylogenetic tree are supported by 1.00 Bayesian inference posterior probability.

In this study, 19 species from Caesalpinioideae and two outgroups were analyzed based on 73 protein-coding genes of their chloroplast genomes. The total concatenated alignment length from the 73 protein-coding genes was 60,736 bp. The reconstructed phylogeny indicated that Caesalpinioideae was paraphyletic and that the species from tribe Mimoseae (*Adenanthera microsperma, Dichrostachys cinerea*, *Leucaena trichandra*, *Parkia javanica*, *Piptadenia communis* and *S. adstringens*) were deemed non-monophyletic (Fig. 7). All nodes were strongly supported, given the Bayesian posterior probability (Fig. 7). These results are consistent with those from Wang *et al*. (2017) and support the new classification system proposed for the Leguminosae^21^.

## Conclusion

In this work, we assemble the complete chloroplast genome of *S. adstringens* with 162,169 bp. Genome gene contents and orientation are similar to those found in the chloroplast genome of other Mimosoid (Leguminosae) species. This study also revealed the distribution and location of repeated structures and microsatellites along the chloroplast genome of *S. adstringens*. We also generated important genomic resources for Mimosoid group. Moreover, the Ka/Ks ratio was lower in the LSC region compared to SSC region. As expected, the comparison with other five Mimosoid species revealed that the coding regions are more conserved than non-coding regions, and IRs more conserved than LSC and SSC regions. Finally, the phylogenetic relationships built for 19 species of Caesalpinioideae, including the new data from *S. adstringens* and two outgroups, were fully resolved with high supports based on 73 conserved protein-coding genes. The maximum credibility tree revealed that the tribe Mimoseae is paraphyletic, consistent with the new classification proposed for the Leguminosae.

## Supplementary information

Supplementary information

## Data Availability

The complete chloroplast sequence generated and analyzed during the current study are available in GenBank, https://www.ncbi.nlm.nih.gov (accession numbers are described in the text).
