# Supplementary material for: The complete chloroplast genome of Stryphnodendron adstringens (Leguminosae - Caesalpinioideae): comparative analysis with related Mimosoid species
Source: Sci Rep. 2019 Oct 2;9:14206. doi: 10.1038/s41598-019-50620-3 (PMC6775074; doi:10.1038/s41598-019-50620-3)
Supplement: Supplementary file 1 — Supplementary information [file 41598_2019_50620_MOESM1_ESM.docx]

**Scientific Reports**

**ELECTRONIC SUPPLEMENTARY MATERIAL**

**The complete chloroplast genome of *Stryphnodendron adstringens* (Leguminosae - Caesalpinioideae): comparative analysis with related Mimosoid species**

**Ueric José Borges de Souza^1^, Rhewter Nunes^1^, Cíntia Pelegrineti Targueta^1^, José Alexandre Felizola Diniz-Filho^2^ and Mariana Pires de Campos Telles^1,3*^**

^1^Laboratório de Genética & Biodiversidade, Departamento de Genética, Instituto de Ciências Biológicas - UFG, Goiânia, 74690-900, Brazil.

^2^Laboratório de Ecologia Teórica e Síntese, Departamento de Ecologia, Instituto de Ciências Biológicas - UFG, Goiânia, 74690-900, Brazil.

^3^Escola de Ciências Agrárias e Biológicas, PUC-GO, Goiânia, Brazil.

These authors contributed equally to this work.

*Correspondence should be addressed to M.P.C.T (e-mail: [tellesmpc@gmail.com](mailto:tellesmpc@gmail.com))

**
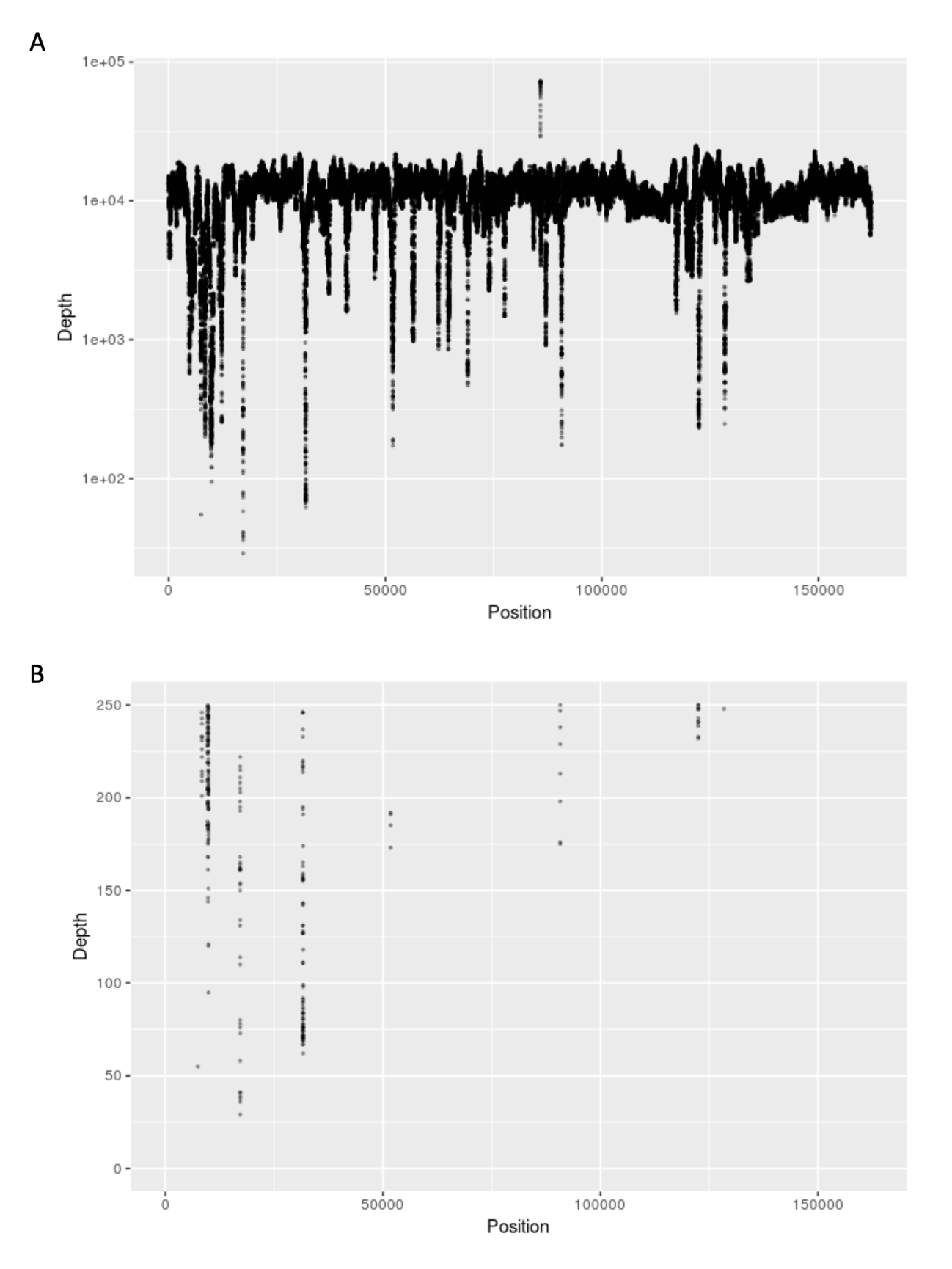
**

**Figure S1.** Genome sequencing coverage distribution in *S. adstringens* chloroplast genome. A) Logarithm scale of all reads that aligned in the genome; B) Zoom in coverage window between 0 and 250 X (excluding values over 250).


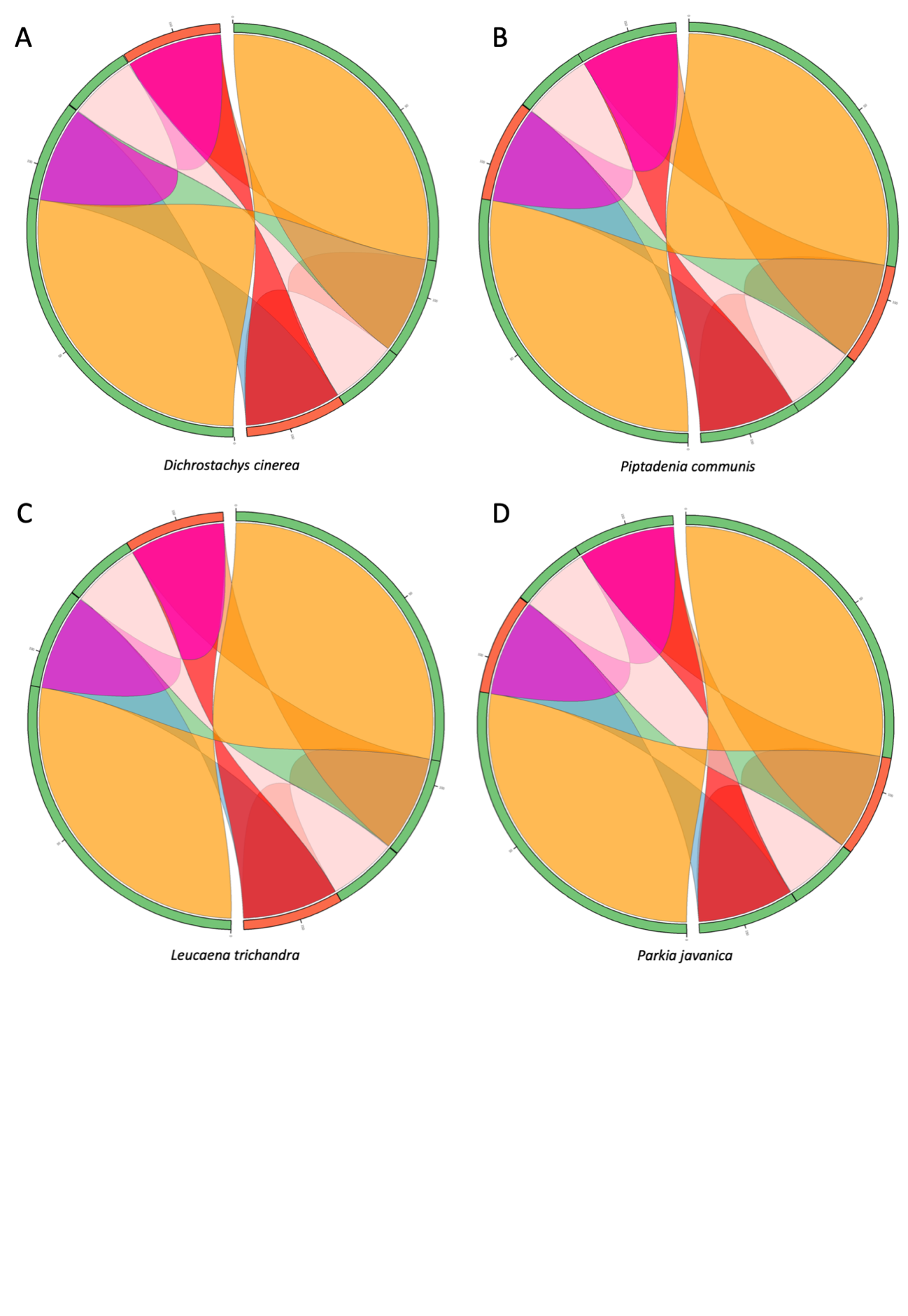


**Figure S2.** Pairwise circular genome comparison between chloroplast chromosomes of *S. adstringens* (left chromosome) and A) *D. cinerea*; B) *P. communis*; C) *L. trichandra* and D) *P. javanica*. The orange bar on the chromosomes represents one of the inverted repeat portions in each chloroplast genome.

**Table S1.** Base composition of the *S. adstringens* chloroplast genome.

| **Regions** | **A (%)** | **T (U) (%)** | **C (%)** | **G (%)** | **Length (bp)** |  |
| --- | --- | --- | --- | --- | --- | --- |
| **Genome** | 31.6 | 32.5 | 18.3 | 17.6 | 162,169 |  |
| **LSC** | 32.6 | 34.1 | 17.1 | 16.1 | 91,045 |  |
| **SSC** | 34.8 | 35.2 | 15.7 | 14.3 | 19,014 |  |
| **IR** | 28.5 | 28.8 | 20.6 | 22.1 | 26,055 |  |
| **Protein coding genes** | 30.8 | 31.8 | 17.4 | 20.0 | 78,739 |  |
| **1st position** | 32.1 | 38.0 | 13.7 | 15.9 | 26,247 |  |
| **2nd position** | 30.9 | 24.0 | 18.5 | 26.4 | 26,247 |  |
| **3rd position** | 29.5 | 33.0 | 20.0 | 17.6 | 26,247 |  |

**Table S2**. Genes with intron in the *S. adstringens* chloroplast genome, including the exon and intron lenght.

| **Gene name** | **Location** | **Exon I**  **(bp)** | **Intron I**  **(bp)** | **Exon II**  **(bp)** | **Intron II**  **(bp)** | **Exon III**  **(bp)** |
| --- | --- | --- | --- | --- | --- | --- |
| *rps16* | LSC | 40 | 937 | 242 | - | - |
| *atpF* | LSC | 145 | 741 | 407 | - | - |
| *rpoC1* | LSC | 432 | 809 | 1,617 | - | - |
| *petB* | LSC | 6 | 799 | 642 | - | - |
| *petD* | LSC | 8 | 745 | 475 | - | - |
| *rpl16* | LSC | 399 | 1,107 | 9 | - | - |
| *rpl2* | IR | 393 | 662 | 435 | - | - |
| *ndhB* | IR | 777 | 685 | 756 | - | - |
| *ndhA* | SSC | 553 | 1,475 | 539 | - | - |
| *ycf3* | LSC | 124 | 721 | 230 | 754 | 153 |
| *clpP* | LSC | 69 | 803 | 291 | 574 | 228 |
| *rps12* | LSC | 114 |  | 232 | 536 | 26 |
| *trnK-UUU* | LSC | 35 | 2,558 | 37 | - | - |
| *trnG-UCC* | LSC | 23 | 721 | 49 | - | - |
| *trnL-UAA* | LSC | 37 | 513 | 50 | - | - |
| *trnV-UAC* | LSC | 34 | 615 | 39 | - | - |
| *trnA-UGC* | IR | 38 | 802 | 35 | - | - |
| *trnI-GAU* | IR | 42 | 948 | 35 | - | - |

**Table S3**. Foward, Reverse and palindromic repeat sequences in the S. adstringens chloroplast genome.

| No. | Type | Repeat length (bp) | Location | Region | Repeat unit |
| --- | --- | --- | --- | --- | --- |
| 1 | F | 30 | IGS (*trnQ-UUG - psbK*) | LSC | TTATTATTAATATTAATTCTATAATATAAT |
| 2 | F | 31 | IGS (*trnP-UGG - psaJ*) | LSC | TAATAATAAGAATAAAGAATAAGAATAAAGA |
| 3 | F | 32 | IGS (*atpH - atpI*) | LSC | TTAATTTATATATTATTATTAATTTATAATTT |
| 4 | F | 32 | IGS (*ndhC - trnV-UAC*) | LSC | GTTATTTAGTATTTAATAGTATTAGTAAGTAT |
| 5 | F | 32 | IGS (*trnR-ACG - trnN-GUU*) | IR | AAAAAGCAAAAAAGATATGCGGATTAGGGCAT |
| 6 | F | 32 | IGS (*trnR-ACG - trnN-GUU*) | IR | AATATAGTATGCCCTAATCCGCATATCTTTTT |
| 7 | F | 33 | IGS (*trnS-GCU - trnG-UCC*) | LSC | TAAACAATATTCTATTTATAGAAAAAAATATTC |
| 8 | F | 33 | *ndhA* (intron) | SSC | TATAAAGAAGAAAAGAAATAAAGACGAAAAGAA |
| 9 | F | 34 | IGS (*accD - psaI*) | LSC | TTTTTATTATATTATTTCTATTTTATTATATTAT |
| 10 | F | 35 | IGS (*atpH - atpI*) | LSC | TTATTATATATATATATTATATATTATTATATATT |
| 11 | F | 35 | IGS (*ndhF - rpl32*) | SSC | TATATAATATAAAAAGGATAGTTTTTTTGATATAA |
| 12 | F | 36 | IGS (*ccsA - ndhD*) | SSC | TAATAAAATACAATAGATAATAAAATAAAATACAAT |
| 13 | F | 36 | IGS (*rps3 - rps19*) | LSC | TCTTTGATACTCTTCTTTCCTTTCATACTCTTCTTC |
| 14 | F | 37 | *ndhA* (intron) | SSC | CTATATCTATATATTATATATTTCTATATTATATCTA |
| 15 | F | 39 | *ycf3* (intron), *ndhA*(intron), IGS(*rps12* - *trnV*-*GAC*) | LSC/SSC/IR | CCAGAACCGTACGTGAGATTTTCATCTCATACGGCTCCT |
| 16 | F | 41 | IGS (*trnP-UGG - psaJ*) | LSC | TAATAAGAATAAAGAATAAGAATAAAGAATAAGAATAAAGA |
| 17 | F | 41 | *psaB* | LSC | ATGCAATAGCTAAATGATGATGAGCAATATCAGTCAGCCAT |
| 18 | F | 42 | IGS (*rps12 - trnV-GAC*) | IR | CTACAGAACCGTACATGAGATTTTCACCTCATACGGCTCCTC |
| 19 | F | 43 | *rpl16* (intron) | LSC | ACTACAGAACCGTACATGAGATTTTCACCTCATACGGCTCCTC |
| 20 | F | 43 | IGS (*rrn5 - trnR-ACG*) | IR | CTCATTCTTATTACTTTTTCATATTGAAAAAGTAATAAGAATG |
| 21 | F | 52 | *rpl16* (intron), *ndhA* (intron) | LSC/SSC | TCCATCTCTACTACAGAACCGTACATGAGATTTTCACCTCATACGGCTCCTC |
| 22 | F | 128 | IGS (*trnC-GCA - petN*) | LSC | TTAGAATATAAAATATAAATAGAATATAAAATATAAATAGAATATAAAATATAAATAGAATATAAAATATAAATAGAATATAAAATATAAATAGAATATAAAATATAAATAGAATATAAAATATAAAT |
| 23 | R | 30 | IGS (*psbZ* - *trnG*-*UCC*) | LSC | ATAGATATAGATAGATATAGATAGATATAG |
| 24 | R | 38 | IGS (*rbcl* - *accD*) | LSC | CTATTTTATATTTCTATATATTTTTATATTTCTATATA |
| 25 | P | 30 | IGS (*trnQ-UUG* - *psbK*) | LSC | TTAATAATTTAATTAATTAATTAAATTATT |
| 26 | P | 31 | IGS (*trnQ*-*UUG* - *psbK*) | LSC | ATTAATTCTATAATATAATTAGAATATTAAT |
| 27 | P | 31 | IGS (*trnT*-*UGU* - *trnL*-*UAA*) | LSC | ATATATCTAAATAAATAGTTATCTATAAAAT |
| 28 | P | 31 | IGS (*trnK*-*UUU* - *rps16*) | LSC | GGAACGGGACTAAATGGATCCATTTAGTCCC |
| 29 | P | 32 | IGS (*rps3* - *rps19*) | LSC | AATAATAAATATTTATATATTAATATTTATTA |
| 30 | P | 32 | IGS (*trnR*-*ACG* - *trnN*-*GUU*) | IR | AAAAAGCAAAAAAGATATGCGGATTAGGGCAT |
| 31 | P | 35 | *trnS*-*GCU* | LSC | GGGAAAGAGAGGGATTCGAACCCTCGGTACGAAAA |
| 32 | P | 37 | IGS (*ccsA* - *ndhD*) | SSC | AATTTTATTAAAATTAATTAAATTAAATTTAATAAAA |
| 33 | P | 39 | *ycf3*(intron) | LSC | CCAGAACCGTACGTGAGATTTTCATCTCATACGGCTCCT |
| 34 | P | 41 | IGS (*trnQ*-*UUG* - *psbK*) | LSC | TTTTATTAATATTAATTCTATAATATAATTAGAATATTAAT |
| 35 | P | 41 | IGS (*accD* - *psaI*) | LSC | ATTTAGAATAGTATATTTAGAATCTAATTATACTATTCTAA |
| 36 | P | 42 | *ndhA* (intron) | SSC | TTACAGAACCGTACATGAGATTTTCACCTCATACGGCTCCTC |
| 37 | P | 43 | *rpl16* (intron), IGS (*trnV*-*GAC* - *rps12*) | LSC | ACTACAGAACCGTACATGAGATTTTCACCTCATACGGCTCCTC |
| 38 | P | 43 | IGS (*rrn5* - *trnR*-*ACG*) | IR | CTCATTCTTATTACTTTTTCATATTGAAAAAGTAATAAGAATG |
| 39 | P | 43 | IGS (*trnR*-*ACG* - *rrn5*) | IR | TTCATTCTTATTACTTTTTCAATATGAAAAAGTAATAAGAATG |
| 40 | P | 44 | IGS (*psbT* - *psbN*) | LSC | ATTGAAGTAATGAGCCTACCAATATTGGCAGGCTCATTACTTCA |
| 41 | P | 49 | IGS (*petN* - *psbM*) | LSC | AGATAGTATGGTAGAAAGAAATAAAATCTATTTCTTTCTACCATACTAT |
| 42 | P | 60 | IGS (*rbcl* - *accD*) | LSC | AATTTCAATTCTATAATATTCAATTCTATTATTATAGAATTGAATATTATAGAATTGAAA |

P: means palindromic, F: means forward, R: means reverse and IGS: means intergenic spacers.

| Table S4. Distribution of simple sequence repeat (SSR) in the S. adstringens chloroplast genome. | | | | | | |
| --- | --- | --- | --- | --- | --- | --- |
| No. SSR | **SSR type** | **SSR** | **Size** | **Start** | **End** | **Location** |
| 1 | p1 | (T)10 | 10 | 215 | 224 | LSC |
| 2 | p3 | (CAG)4 | 12 | 1065 | 1076 | LSC (psbA) |
| 3 | p4 | (TTCA)3 | 12 | 1721 | 1732 | LSC |
| 4 | p1 | (T)13 | 13 | 2067 | 2079 | LSC |
| 5 | p1 | (A)14 | 14 | 3897 | 3910 | LSC |
| 6 | p1 | (A)13 | 13 | 4439 | 4451 | LSC |
| 7 | p1 | (A)11 | 11 | 4553 | 4563 | LSC |
| 8 | p1 | (A)13 | 13 | 4990 | 5002 | LSC |
| 9 | p2 | (AT)6 | 12 | 5302 | 5313 | LSC |
| 10 | c | (G)10agataagagagaaaaggattatataaatctacataaaagtcatccacaccctctttttttcttttttga(ATTT)3 | 91 | 5900 | 5990 | LSC |
| 11 | c | (T)12cttattctattctttcccg(A)10 | 41 | 6399 | 6439 | LSC (*rps16) |
| 12 | p1 | (T)10 | 10 | 6833 | 6842 | LSC (*rps16) |
| 13 | p1 | (A)13 | 13 | 7674 | 7686 | LSC |
| 14 | c | (ATT)4tcttatttc(T)14attattattttattaattaataat(TTAA)4attattattaatattaattctataatataattttattaatattaattctataatataattagaatattaattaatagtataaatcataaatgaaat(A)10ttcaattaaaaattcatttattagaaaattcaga(AT)6aattaataat(AATAGA)3 | 255 | 8357 | 8611 | LSC |
| 15 | p1 | (A)13 | 13 | 8953 | 8965 | LSC |
| 16 | p4 | (CAAA)3 | 12 | 9386 | 9397 | LSC |
| 17 | c | (AT)6ttctataatattctatagatattctatagaataataaaattagaatactaataataaaattagaatactaata(AAT)4atttcagaatttatatattaat(ATTA)3 | 131 | 9635 | 9765 | LSC |
| 18 | c | (A)11tacttgttattgaaacaagatcaaacataagacataagaatgtcatttcttattcctctttctttttttccgctaaaatatctaaaagaaaaaag(A)13 | 119 | 10459 | 10577 | LSC |
| 19 | c | (AT)6tttttagctcgatgaaaatcaaatgcttttcctaaggattctcttaaatagaaatagagaacgaagtaactag(A)18 | 103 | 11234 | 11336 | LSC |
| 20 | p1 | (T)12 | 12 | 14736 | 14747 | LSC (*atpF) |
| 21 | p1 | (T)10 | 10 | 14911 | 14920 | LSC (*atpF) |
| 22 | p3 | (TTA)4 | 12 | 15477 | 15488 | LSC |
| 23 | p1 | (A)14 | 14 | 16346 | 16359 | LSC |
| 24 | p1 | (T)10 | 10 | 16972 | 16981 | LSC |
| 25 | c | (TA)6ttatatattattatat(ATTAA)3 | 43 | 17214 | 17256 | LSC |
| 26 | c | (T)10acaattccctggtaatcttttttacttttagacaaaatcgtatacttattttatttagaccttatttgcatctttc(TTATT)3 | 101 | 17421 | 17521 | LSC |
| 27 | c | (T)11caaat(A)10gaatttg(A)12 | 45 | 19338 | 19382 | LSC |
| 28 | p1 | (T)13 | 13 | 19681 | 19693 | LSC (rpoC2) |
| 29 | c | (T)12accgttccgagtggtatcaagatgccgctgtgtcgggatatcttatccatctctccaggaaaatagatatccccagaaaagatttttaattcaatcc(T)10 | 119 | 21486 | 21604 | LSC (rpoC2) |
| 30 | p1 | (T)10 | 10 | 22129 | 22138 | LSC (rpoC2) |
| 31 | p2 | (TA)5 | 10 | 22973 | 22982 | LSC (rpoC2) |
| 32 | c | (A)13gaagtc(T)12 | 31 | 26103 | 26133 | LSC (*rpoC1) |
| 33 | p1 | (T)10 | 10 | 29375 | 29384 | LSC (rpoB) |
| 34 | p1 | (T)10 | 10 | 30113 | 30122 | LSC |
| 35 | p1 | (A)10 | 10 | 31165 | 31174 | LSC |
| 36 | p4 | (TTCT)3 | 12 | 31750 | 31761 | LSC |
| 37 | p2 | (TA)5 | 10 | 31887 | 31896 | LSC |
| 38 | p4 | (TTAA)3 | 12 | 32404 | 32415 | LSC |
| 39 | p1 | (T)11 | 11 | 33886 | 33896 | LSC |
| 40 | p2 | (TG)5 | 10 | 34122 | 34131 | LSC |
| 41 | p1 | (A)14 | 14 | 35274 | 35287 | LSC |
| 42 | p1 | (T)11 | 11 | 35996 | 36006 | LSC |
| 43 | c | (A)15tagactttggagttttttttattaatctgaaagaaaggaaaatataacaaagaagacaataccaataaaag(A)16 | 102 | 36942 | 37043 | LSC |
| 44 | c | (TA)5catgcaggatccagcatgcccctttgtcaagt(A)10 | 52 | 40405 | 40456 | LSC |
| 45 | c | (ACTAT)3aatactataactatatataactataat(TATAA)3 | 57 | 47494 | 47550 | LSC |
| 46 | c | (T)10ctcgagtt(A)15 | 33 | 47896 | 47928 | LSC (*ycf3) |
| 47 | p1 | (T)10 | 10 | 50848 | 50857 | LSC |
| 48 | p2 | (TA)5 | 10 | 50981 | 50990 | LSC |
| 49 | p3 | (AAT)4 | 12 | 51856 | 51867 | LSC |
| 50 | p1 | (A)11 | 11 | 52224 | 52234 | LSC |
| 51 | p1 | (T)11 | 11 | 53472 | 53482 | LSC |
| 52 | p2 | (AT)5 | 10 | 56199 | 56208 | LSC |
| 53 | p1 | (T)15 | 15 | 56362 | 56376 | LSC |
| 54 | p4 | (TACA)3 | 12 | 56489 | 56500 | LSC |
| 55 | p1 | (T)10 | 10 | 59779 | 59788 | LSC (atpB) |
| 56 | p1 | (T)10 | 10 | 60222 | 60231 | LSC |
| 57 | p1 | (A)14 | 14 | 60393 | 60406 | LSC |
| 58 | p3 | (TTA)4 | 12 | 62856 | 62867 | LSC |
| 59 | p4 | (AATA)3 | 12 | 64584 | 64595 | LSC |
| 60 | p1 | (T)13 | 13 | 64737 | 64749 | LSC |
| 61 | p1 | (A)12 | 12 | 65311 | 65322 | LSC |
| 62 | c | (A)10tgaactgaaaactgaatggaatgctttctt(A)13 | 53 | 65893 | 65945 | LSC |
| 63 | p1 | (T)14 | 14 | 68432 | 68445 | LSC |
| 64 | c | (A)10tagaaaaaataaatagatactatatac(TA)5 | 47 | 69039 | 69085 | LSC |
| 65 | p1 | (A)10 | 10 | 70797 | 70806 | LSC |
| 66 | p4 | (TGAT)3 | 12 | 71479 | 71490 | LSC |
| 67 | c | (TA)5gcataatc(TA)5gaactctcatc(AT)5 | 49 | 72289 | 72337 | LSC |
| 68 | p3 | (ATA)4 | 12 | 72642 | 72653 | LSC |
| 69 | p1 | (T)10 | 10 | 73160 | 73169 | LSC |
| 70 | p2 | (TA)5 | 10 | 73924 | 73933 | LSC |
| 71 | p1 | (A)13 | 13 | 74461 | 74473 | LSC (rps18) |
| 72 | p1 | (T)14 | 14 | 75780 | 75793 | LSC |
| 73 | p1 | (T)11 | 11 | 76780 | 76790 | LSC (*clpP) |
| 74 | c | (A)10tcaatgtgtagatttcaaccctctttc(T)12 | 49 | 77417 | 77465 | LSC (*clpP) |
| 75 | c | (T)11caaggagttagaagttataataaatcgaaagattttgattctttcaaactgctgcttatcc(T)12 | 84 | 81377 | 81460 | LSC (*petB) |
| 76 | c | (T)12ag(A)13 | 27 | 84249 | 84275 | LSC |
| 77 | c | (T)23gtgcatccggtcaagccccc(T)20 | 63 | 85878 | 85940 | LSC |
| 78 | p3 | (ATT)4 | 12 | 87059 | 87070 | LSC |
| 79 | c | (A)14gaaatattgtgtatc(A)10 | 39 | 87709 | 87747 | LSC |
| 80 | p1 | (T)12 | 12 | 89093 | 89104 | LSC (*rpl16) |
| 81 | c | (ATTA)3a(TAC)4 | 25 | 89224 | 89248 | LSC (*rpl16) |
| 82 | p1 | (T)10 | 10 | 90173 | 90182 | LSC |
| 83 | p1 | (T)10 | 10 | 90371 | 90380 | LSC |
| 84 | p1 | (T)10 | 10 | 91165 | 91174 | IRA |
| 85 | p5 | (AATGG)3 | 15 | 101485 | 101499 | IRA |
| 86 | p2 | (AG)5 | 10 | 115211 | 115220 | IRA |
| 87 | p5 | (TTAAT)3 | 15 | 117210 | 117224 | SSC |
| 88 | p1 | (T)10 | 10 | 118022 | 118031 | SSC (ndhF) |
| 89 | p1 | (A)14 | 14 | 119489 | 119502 | SSC |
| 90 | p1 | (T)12 | 12 | 119714 | 119725 | SSC |
| 91 | p1 | (A)11 | 11 | 120298 | 120308 | SSC |
| 92 | p2 | (TA)5 | 10 | 120715 | 120724 | SSC |
| 93 | p1 | (T)10 | 10 | 120843 | 120852 | SSC |
| 94 | p1 | (T)11 | 11 | 122704 | 122714 | SSC |
| 95 | p1 | (T)13 | 13 | 130963 | 130975 | SSC |
| 96 | p1 | (T)11 | 11 | 132548 | 132558 | SSC (ycf1) |
| 97 | p1 | (T)11 | 11 | 132755 | 132765 | SSC (ycf1) |
| 98 | p1 | (T)12 | 12 | 132906 | 132917 | SSC (ycf1) |
| 99 | p1 | (T)11 | 11 | 133696 | 133706 | SSC (ycf1) |
| 100 | p1 | (T)10 | 10 | 134082 | 134091 | SSC (ycf1) |
| 101 | c | (T)13agcttcggaatccaccttttcatgttctcgaaataaataaagtcctttaaaattgaaattggatactgattttgcagaaaattgactgatcaagtt(A)10 | 119 | 135144 | 135262 | SSC (ycf1) |
| 102 | p1 | (T)11 | 11 | 135967 | 135977 | SSC (ycf1) |
| 103 | p2 | (CT)5 | 10 | 137994 | 138003 | IRB |
| 104 | p5 | (TTCCA)3 | 15 | 151713 | 151727 | IRB |
| 105 | p1 | (A)10 | 10 | 162040 | 162049 | IRB |

Gene name in parenthesis indicates SSR that is coded within the CDS of the respective gene

* SSR in the intronic region of the respective gene in parenthesis

**Table S5.** The Ka, Ks and Ka/Ks ratio of Mimosoid chloroplast genome for individual genes and region.

| Gene groups | gene | Ka | Ks | Ka/Ks | Region |
| --- | --- | --- | --- | --- | --- |
| Large subunit of ribosomal proteins | *rpl33* | 0.00652 | 0.0445 | 0.146517 | LSC |
|  | *rpl20* | 0.01379 | 0.02411 | 0.571962 | LSC |
|  | *rpl36* | 0.00000 | 0.03904 | 0.00000 | LSC |
|  | *rpl14* | 0.00120 | 0.04091 | 0.029333 | LSC |
|  | *rpl16* | 0.00111 | 0.06961 | 0.015946 | LSC |
|  | *rpl2* | 0.00108 | 0.00989 | 0.109201 | IR |
|  | *rpl23* | 0.00156 | 0.00000 | 0.00000 | IR |
|  | *rpl32* | 0.01046 | 0.09263 | 0.112922 | SSC |
| Small subunit of ribosomal proteins | *rps16* | 0.02246 | 0.00574 | 3.912892 | LSC |
|  | *rps2* | 0.00486 | 0.01590 | 0.305660 | LSC |
|  | *rps14* | 0.00577 | 0.01520 | 0.379605 | LSC |
|  | *rps4* | 0.00291 | 0.02525 | 0.115248 | LSC |
|  | *rps18* | 0.00652 | 0.04801 | 0.135805 | LSC |
|  | *rps11* | 0.00217 | 0.03277 | 0.066219 | LSC |
|  | *rps8* | 0.00554 | 0.05103 | 0.108564 | LSC |
|  | *rps3* | 0.00567 | 0.03538 | 0.16026 | LSC |
|  | *rps19* | 0.00598 | 0.04149 | 0.144131 | LSC |
|  | *rps7* | 0.00343 | 0.00889 | 0.385827 | IR |
|  | *rps12* | 0.00000 | 0.01458 | 0.000000 | IR |
|  | *rps15* | 0.01777 | 0.0375 | 0.473867 | SSC |
| DNA-dependent RNA polymerase | *rpoC2* | 0.00912 | 0.03179 | 0.286883 | LSC |
|  | *rpoC1* | 0.00497 | 0.02452 | 0.202692 | LSC |
|  | *rpoB* | 0.00423 | 0.02317 | 0.182564 | LSC |
|  | *rpoA* | 0.00903 | 0.04328 | 0.208641 | LSC |
| Photosystem I | *psaB* | 0.00138 | 0.02978 | 0.046340 | LSC |
|  | *psaA* | 0.00175 | 0.0248 | 0.070565 | LSC |
|  | *psaI* | 0.02987 | 0.05379 | 0.555308 | LSC |
|  | *psaJ* | 0.00338 | 0.05941 | 0.056893 | LSC |
|  | *psaC* | 0.00000 | 0.05761 | 0.000000 | SSC |
| Photosystem II | *psbA* | 0.00231 | 0.02972 | 0.077725 | LSC |
|  | *psbK* | 0.00980 | 0.04920 | 0.199187 | LSC |
|  | *psbI* | 0.00416 | 0.02023 | 0.205635 | LSC |
|  | *psbM* | 0.00000 | 0.04284 | 0.000000 | LSC |
|  | *psbD* | 0.00041 | 0.02315 | 0.017711 | LSC |
|  | *psbC* | 0.00062 | 0.02620 | 0.023664 | LSC |
|  | *psbZ* | 0.00000 | 0.02609 | 0.000000 | LSC |
|  | *psbJ* | 0.00000 | 0.00000 | 0.000000 | LSC |
|  | *psbL* | 0.00416 | 0.02023 | 0.205635 | LSC |
|  | *psbF* | 0.00000 | 0.01118 | 0.000000 | LSC |
|  | *psbE* | 0.00175 | 0.01173 | 0.149190 | LSC |
|  | *psbB* | 0.00112 | 0.02898 | 0.038647 | LSC |
|  | *psbT* | 0.01757 | 0.00000 | 0.000000 | LSC |
|  | *psbN* | 0.00000 | 0.01047 | 0.000000 | LSC |
|  | *psbH* | 0.02040 | 0.01214 | 1.680395 | LSC |
| NADH dehydrogenase | *ndhJ* | 0.00182 | 0.02749 | 0.066206 | LSC |
|  | *ndhK* | 0.00641 | 0.02938 | 0.218176 | LSC |
|  | *ndhC* | 0.00928 | 0.03154 | 0.294230 | LSC |
|  | *ndhB* | 0.00162 | 0.00179 | 0.905028 | IR |
|  | *ndhF* | 0.01270 | 0.03726 | 0.340848 | SSC |
|  | *ndhD* | 0.00725 | 0.03343 | 0.216871 | SSC |
|  | *ndhE* | 0.00419 | 0.05561 | 0.075346 | SSC |
|  | *ndhG* | 0.00721 | 0.04555 | 0.158288 | SSC |
|  | *ndhI* | 0.00324 | 0.03583 | 0.090427 | SSC |
|  | *ndhA* | 0.00963 | 0.01685 | 0.571513 | SSC |
|  | *ndhH* | 0.00388 | 0.03389 | 0.114488 | SSC |
| Cytochrome b/f complex | *petN* | 0.00512 | 0.01621 | 0.315854 | LSC |
|  | *petA* | 0.00614 | 0.03184 | 0.192839 | LSC |
|  | *petL* | 0.00000 | 0.03487 | 0.000000 | LSC |
|  | *petG* | 0.00000 | 0.01184 | 0.000000 | LSC |
|  | *petB* | 0.00137 | 0.04232 | 0.032372 | LSC |
|  | *petD* | 0.00092 | 0.02175 | 0.042299 | LSC |
| ATP synthase | *atpA* | 0.00159 | 0.0428 | 0.037150 | LSC |
|  | *atpF* | 0.00675 | 0.02774 | 0.243331 | LSC |
|  | *atpH* | 0.00000 | 0.02566 | 0.000000 | LSC |
|  | *atpI* | 0.00370 | 0.02032 | 0.182087 | LSC |
|  | *atpE* | 0.00530 | 0.02428 | 0.218287 | LSC |
|  | *atpB* | 0.00089 | 0.03743 | 0.023778 | LSC |
| RubisCo large subunit | *rbcL* | 0.01105 | 0.04678 | 0.236212 | LSC |
| Maturase K | *matK* | 0.01494 | 0.02608 | 0.572853 | LSC |
| Envelope membrane protein | *cemA* | 0.00561 | 0.02283 | 0.245729 | LSC |
| Subunit of acetyl-CoAcarboxylase | *accD* | 0.01713 | 0.02936 | 0.583447 | LSC |
| C-type cytochrome synthesis gene | *ccsA* | 0.01181 | 0.02449 | 0.482238 | SSC |
| Protease | *clpP* | 0.02109 | 0.01255 | 1.680478 | LSC |
| Conserved hypothetical chloroplast open reading frames | *ycf3* | 0.00522 | 0.00869 | 0.600690 | LSC |
|  | *ycf4* | 0.00380 | 0.02053 | 0.185095 | LSC |
|  | *ycf2* | 0.00383 | 0.00551 | 0.695100 | IR |
|  | *ycf1* | 0.03534 | 0.04383 | 0.806297 | SSC |

*Ka* non-synonymous substitution, *Ks* synonymous, LSC Large Single Copy, SSC Small Single Copy, IR Inverted Repeat.

**Table S6**. Accession number and sampled chloroplast genomes obtained from GenBank.

| **Species** | **Accession** | **Genome Size** |
| --- | --- | --- |
| **Caesalpinioideae** | | |
| *Acacia dealbata* | NC_034985 | 174217 bp |
| *Acacia ligulata* | NC_026134 | 174233 bp |
| *Adenanthera microsperma* | NC_034986 | 159389 bp |
| *Albizia odoratissima* | NC_034987 | 174861 bp |
| *Archidendron lucyi* | NC_034988 | 176870 bp |
| *Dichrostachys cinerea* | NC_035346 | 161240 bp |
| *Faidherbia albida* | NC_035347 | 175646 bp |
| *Inga leiocalycina* | NC_028732 | 175489 bp |
| *Leucaena trichandra* | NC_028733 | 164692 bp |
| *Pararchidendron pruinosum* | NC_035348 | 176692 bp |
| *Parkia javanica* | NC_034989 | 161681 bp |
| *Piptadenia communis* | NC_034990 | 162552 bp |
| *Pithecellobium flexicaule* | NC_034991 | 178887 bp |
| *Samanea saman* | NC_034992 | 176717 bp |
| *Senegalia laeta* | NC_036736 | 162754 bp |
| *Senna tora* | NC_030193 | 162426 bp |
| *Vachellia flava* | NC_036734 | 165829 bp |
| *Vachellia seyal* | NC_036735 | 165383 bp |
|  | **Outgroup** |  |
| *Cucumis sativus* | NC_007144 | 155293 bp |
| *Fragaria vesca* | NC_015206 | 155691 bp |

**Table S7**. Models, Gamma Distribution, Proportion of Invariant Sites and Kappa, as Estimated by jModelTest for Each Gene Alignment.

| **Gene** | **Model** | **Gamma** | **Invariant sites** | **Kappa** |
| --- | --- | --- | --- | --- |
| *atpA* | GTR+I+G | 0.849 | 0.559 | N/A |
| *atpB* | GTR+G | 0.270 | N/A | N/A |
| *atpE* | HKY+I | N/A | 0.705 | 3,117 |
| *atpF* | GTR+I | N/A | 0.466 | N/A |
| *atpH* | GTR+I | N/A | 0.784 | N/A |
| *atpI* | GTR+G | 0.165 | N/A | N/A |
| *ccsA* | GTR+G | 0.543 | N/A | N/A |
| *cemA* | GTR+G | 1,060 | N/A | N/A |
| *clpP* | GTR+I+G | 0.705 | 0.050 | N/A |
| *matK* | GTR+G | 1,000 | N/A | N/A |
| *ndhA* | GTR+G | 0.390 | N/A | N/A |
| *ndhB* | GTR+I | N/A | 0.808 | N/A |
| *ndhC* | GTR+G | 0.639 | N/A | N/A |
| *ndhD* | GTR+G | 0.420 | N/A | N/A |
| *ndhE* | HKY+G | 0.600 | N/A | 5,017 |
| *ndhF* | GTR+I+G | 0.911 | 0.289 | N/A |
| *ndhG* | GTR+G | 1,063 | N/A | N/A |
| *ndhH* | GTR+I+G | 0.673 | 0.442 | N/A |
| *ndhI* | GTR+I | N/A | 0.648 | N/A |
| *ndhJ* | GTR+G | 0.239 | N/A | N/A |
| *ndhK* | GTR+G | 0.304 | N/A | N/A |
| *petA* | GTR+G | 0.213 | N/A | N/A |
| *petB* | HKY+I | N/A | 0.763 | 6,439 |
| *petD* | HKY+G | 0.118 | N/A | 4,329 |
| *petG* | HKY+I | N/A | 0.765 | 4,481 |
| *petL* | HKY+I | N/A | 0.625 | 2,962 |
| *petN* | GTR | N/A | N/A | N/A |
| *psaA* | GTR+I+G | 0.832 | 0.563 | N/A |
| *psaB* | GTR+G | 0.096 | N/A | N/A |
| *psaC* | GTR+I | N/A | 0.760 | N/A |
| *psaI* | HKY+G | 0.504 | N/A | 5,257 |
| *psaJ* | HKY+I | N/A | 0.421 | 4,913 |
| *psbA* | GTR+G | 0.120 | N/A | N/A |
| *psbB* | GTR+I+G | 0.677 | 0.586 | N/A |
| *psbC* | GTR+I+G | 0.762 | 0.579 | N/A |
| *psbD* | GTR+I+G | 0.777 | 0.613 | N/A |
| *psbE* | HKY+I | N/A | 0.805 | 10,311 |
| *psbF* | GTR | N/A | N/A | N/A |
| *psbH* | GTR+G | 0.789 | N/A | N/A |
| *psbI* | HKY | N/A | N/A | 9,224 |
| *psbJ* | GTR+I | N/A | 0.593 | N/A |
| *psbK* | HKY+I | N/A | 0.533 | 4,310 |
| *psbL* | HKY | N/A | N/A | 6,413 |
| *psbM* | HKY+I | N/A | 0.730 | 7,709 |
| *psbN* | HKY | N/A | N/A | 4,153 |
| *psbT* | GTR+I | N/A | 0.608 | N/A |
| *psbZ* | HKY+I | N/A | 0.694 | 10,086 |
| *rbcL* | GTR+I+G | 0.748 | 0.655 | N/A |
| *rpl2* | HKY | N/A | N/A | 4,161 |
| *rpl14* | HKY+G | 0.148 | N/A | 5,142 |
| *rpl16* | GTR+G | 0.203 | N/A | N/A |
| *rpl20* | GTR+I+G | 0.748 | 0.470 | N/A |
| *rpl23* | GTR | N/A | N/A | N/A |
| *rpl32* | HKY+G | 1,119 | N/A | 1,981 |
| *rpl33* | GTR+G | 0.338 | N/A | N/A |
| *rpl36* | HKY+G | 0.041 | N/A | 6,957 |
| *rpoA* | GTR+G | 0.550 | N/A | N/A |
| *rpoB* | GTR+G | 0.293 | N/A | N/A |
| *rpoC1* | GTR+I+G | 0.761 | 0.431 | N/A |
| *rpoC2* | GTR+G | 0.536 | N/A | N/A |
| *rps2* | GTR+I+G | 0.875 | 0.481 | N/A |
| *rps3* | GTR+G | 0.555 | N/A | N/A |
| *rps4* | GTR+I+G | 0.826 | 0.327 | N/A |
| *rps7* | GTR | N/A | N/A | N/A |
| *rps8* | GTR+I | N/A | 0.577 | N/A |
| *rps11* | GTR+G | 0.922 | N/A | N/A |
| *rps14* | HKY+I | N/A | 0.603 | 7,049 |
| *rps15* | GTR+G | 0.943 | N/A | N/A |
| *rps18* | HKY+G | 1,127 | N/A | 2,797 |
| *rps19* | GTR+I+G | 0.800 | 0.411 | N/A |
| *ycf2* | GTR+I+G | 0.893 | 0.560 | N/A |
| *ycf3* | GTR+I | N/A | 0.692 | N/A |
| *ycf4* | GTR+G | 0.522 | N/A | N/A |

GTR = General Time Reversible; HKY85 = Hasegawa, Kishino and Yano (1985);
